# Baseline Nutritional Status and Early Treatment Response in Oropharyngeal Cancer: A Prospective Cohort Study by HPV Status (FIS 19 Study)

**DOI:** 10.3390/nu18132091

**Published:** 2026-06-26

**Authors:** Maryam Choulli, Sara Tous, Gonzalo Peón Peña, Beatriz Cirauqui, Anna Sumarroca, Elisenda Climent, Laia Fontane, Isabel Cots, Jesús Brenes, Marisa Mena, Marc Oliva, Laia Alemany, Ricard Mesia, Lorena Arribas

**Affiliations:** 1 Unit of Molecular Epidemiology and Genetics (UNICEMG), Cancer Epidemiology Research Program, Catalan Institute of Oncology (ICO-IDIBELL), Av. de la Granvia de l’Hospitalet, 199, 08908 L’Hospitalet de Llobregat, Spaingpeon@idibell.cat (G.P.P.);; 2 Faculty of Pharmacy and Food Sciences, University of Barcelona, Campus Diagonal, Av. de Joan XXIII, 27–31, 08028 Barcelona, Spain; 3 Clinical Nutrition Unit, Catalan Institute of Oncology (ICO), Av. de la Granvia de l’Hospitalet, 199, 08908 L’Hospitalet de Llobregat, Spain; larribas@iconcologia.net; 4 Centro de Investigación Biomédica en Red: Epidemiología y Salud Pública (CIBERESP), Instituto de Salud Carlos III, Av. Monforte de Lemos 5, 28029 Madrid, Spain; 5 Medical Oncology Department, Catalan Institute of Oncology (ICO)-Badalona, B-ARGO, CARE Program, Germans Trias i Pujol Research Institute (IGTP), Carretera de Canyet s/n, 08916 Badalona, Spain; 6 Department of Otorhinolaryngology and Head and Neck Surgery, Hospital del Mar Research Institute (IMIM), Hospital del Mar, Passeig Marítim 25–29, 08003 Barcelona, Spain; 7 Endocrinology and Nutrition Unit, Hospital del Mar, Parc de Salut Mar, Passeig Marítim, 25–29, 08003 Barcelona, Spain; 8 Clinical Nutrition Unit, Catalan Institute of Oncology (ICO)-Badalona, Carretera de Can Ruti, 08916 Badalona, Spain; 9 Medical Oncology Department, Catalan Institute of Oncology (ICO), ONCOBELL, Av. de la Granvia de l’Hospitalet 199, 08908 L’Hospitalet de Llobregat, Spain; jesusbrenescastro@gmail.com (J.B.);; 10 Bellvitge Biomedical Research Institute (IDIBELL), Av. de la Granvia de l’Hospitalet 199, 08908 L’Hospitalet de Llobregat, Spain

**Keywords:** oropharyngeal squamous cell carcinoma, human papillomavirus, nutritional assessment, malnutrition, body composition, early treatment response

## Abstract

**Background/Objectives**: Human papillomavirus (HPV) is a well-established prognostic marker in oropharyngeal squamous cell carcinoma (OPSCC); however, the short-term treatment response remains heterogeneous, particularly among HPV-positive patients. Given the high prevalence of malnutrition in head and neck cancer, this study examined whether baseline nutritional status, body composition and functional status were associated with early treatment response in OPSCC according to HPV status. **Methods**: A prospective observational multicenter cohort study of newly diagnosed OPSCC patients eligible for curative-intent treatment was conducted at three tertiary hospitals in Barcelona, Spain. Baseline assessments comprised anthropometry, computed tomography (CT)-based body composition at L3, functional performance tests, systemic inflammatory biomarkers and nutritional diagnosis by the Patient-Generated Subjective Global Assessment (PG-SGA). Early treatment response, assessed around 12 weeks post-therapy, was classified as complete remission (CR) or non-complete remission (NCR). Classification tree analyses were performed separately by HPV status. **Results**: Of 101 enrolled patients, 97 completed post-treatment assessment, of whom 51% were HPV-positive. Among HPV-positive patients, PG-SGA score was the main discriminating variable for early response within the classification tree model, with CR achieved in 74% of patients scoring <6 versus 33% of those scoring ≥6 (AUC 0.68, 95% CI 0.55–0.82). Conversely, Eastern Cooperative Oncology Group Performance Status (ECOG PS) and age were the primary discriminating variables in HPV-negative patients (AUC 0.81, 95% CI 0.70–0.93). In both HPV subgroups, body composition and inflammatory markers were not retained in the analysis once nutritional and functional status were considered. **Conclusions**: PG-SGA-defined nutritional status was associated with early treatment response in HPV-positive patients, while functional status was the main variable retained in HPV-negative patients. These findings support the potential clinical value of standardized nutritional assessment in OPSCC and suggest that early identification of poor nutritional status or functional impairment may help refine supportive care planning at treatment initiation.

## 1. Introduction

The prognostic landscape of oropharyngeal squamous cell carcinoma (OPSCC) has been redefined by the identification of human papillomavirus (HPV) as a key biomarker. HPV-positive disease is consistently associated with improved treatment responses and survival outcomes, leading to its integration into contemporary staging and management systems [1,2,3]. Yet up to 25% of HPV-positive patients still experience poor outcomes, indicating that HPV status alone is insufficient for reliable risk stratification [4]. Beyond HPV, lifestyle-related factors such as tobacco and alcohol consumption contribute to systemic inflammation, treatment toxicity, and nutritional deterioration [5]. In this context, malnutrition, sarcopenia and cancer cachexia are frequent in OPSCC, with prevalence rates ranging from 19% to 69% for sarcopenia [6,7,8,9], and 6% to 70% for cancer cachexia before treatment [10,11]. These conditions are associated with higher treatment toxicity, impaired functional status, and reduced treatment tolerance [12]. Elevated tumor metabolic activity in non-metastatic head and neck cancer further correlates with systemic inflammation, malnutrition and sarcopenia, even after stratification by HPV status, reinforcing the prognostic value of nutritional and inflammatory alterations beyond HPV alone [13].

In our previous work, we explored nutritional and body composition changes in OPSCC according to HPV status [14,15]. At diagnosis, HPV-positive patients had better baseline nutritional status and greater adiposity compared to HPV-negative patients. Nevertheless, both groups experienced similar losses of muscle and fat during treatment, with limited nutritional recovery thereafter. At the same time, HPV status was not independently associated with changes in body composition. Despite these overall trends, considerable heterogeneity was observed within both groups: some HPV-positive patients developed marked nutritional deterioration, whereas some HPV-negative patients remained relatively stable. Even so, previous studies [16,17,18] have reported that long-term feeding tube dependence and late complications tend to be less common in HPV-positive patients.

While these nutritional and functional patterns are well documented, the combined association of baseline nutritional status, body composition, and muscle functionality with early treatment response in OPSCC remains unclear. According to the European Society for Clinical Nutrition and Metabolism (ESPEN), a nutritional diagnosis is recommended, rather than a medical screening alone, for early detection and guidance of interventions in oncology [19]. Integrating computed tomography (CT)-based body composition analysis with functional performance testing may provide a more precise framework for early response stratification in OPSCC.

This prospective multicenter study aimed to explore whether baseline nutritional status, body composition and muscle functionality were associated with early treatment response in OPSCC, according to HPV status.

## 2. Materials and Methods

### 2.1. Study Design and Population

The FIS19 study is a multicenter prospective cohort study focused on patients newly diagnosed with primary OPSCC eligible for curative-intent treatment. Patients were recruited at the Catalan Institute of Oncology (ICO)-Hospitalet and Bellvitge Hospital (HUB), ICO-Badalona and Hospital del Mar in Barcelona, Spain. All centers followed standardized treatment protocols for OPSCC, in accordance with the Spanish Society of Medical Oncology (SEOM) clinical practice guidelines [20]. Patients were eligible if they were candidates for a curative-intent treatment, including chemoradiotherapy (CRT) and bioradiotherapy (cetuximab-based therapy), with or without induction chemotherapy. Eligible patients were systematically identified through multidisciplinary tumor board meetings between December 2021 and June 2024, with follow-up completed in December 2024. Exclusion criteria were metastatic disease at diagnosis, a previous history of treated OPSCC, candidates for primary surgical treatment, or cognitive impairments or language barriers that prevented comprehensive assessment.

Ethical approval was obtained from the Clinical Research Ethics Committee of the hospital (reference number PR296/20), and written informed consent was obtained prior to inclusion. Baseline data included blood samples and Formalin-Fixed, Paraffin-Embedded Tissue (FFPE) tumor blocks for HPV testing. Tobacco and alcohol status were categorized as current, non-regular or former (patients reporting abstinence for >1 year before diagnosis). Smoking was also quantified using the Smokerlyzer^®^ TouchScreen CO-oximeter (Bedfont Scientific Ltd., Harrietsham, UK) [21].

**Figure 1:**
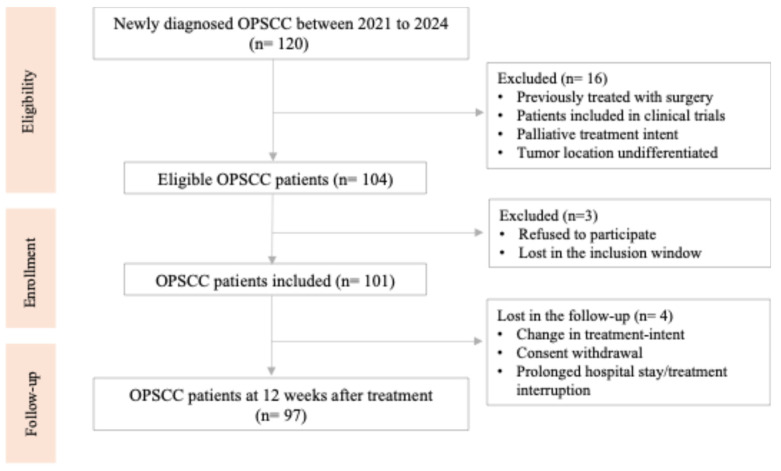
STROBE flowchart of study enrollment.

This study follows the STROBE (Strengthening the Reporting of Observational Studies in Epidemiology) guidelines [22]. A STROBE flowchart (Figure 1) illustrates patient inclusion, exclusions and follow-up. The full checklist is provided in the Supplementary Materials (Table S5).

### 2.2. Sample Size Calculation

The FIS19 study was designed with an a priori sample size calculation of 100 patients, based on survival outcomes, with an alpha risk of 0.10 for a one-tailed test and a power of 0.7. The present work represents an exploratory analysis of this cohort focused on predictors of early treatment response.

### 2.3. Treatment Response Assessment

Early treatment response was defined as the first post-treatment assessment, performed approximately 12 weeks after completion of radiotherapy. The response was assessed by the multidisciplinary tumor board based on clinical examination, flexible nasofibroscopy and PET/CT image findings. Complete remission (CR) was defined as the absence of disease, whereas non-complete remission (NCR) included disease persistence or progression at the time of assessment. Since no patients underwent salvage surgery, the CR status reflects the initial response to radiotherapy-based treatment.

### 2.4. Nutritional and Body Composition Assessment

Nutritional assessment was performed by an oncology dietitian and included anthropometry, and assessment of nutrition-impact symptoms such as odynophagia, dysphagia or anorexia. Percentage weight loss was calculated as the difference between the patient’s usual weight (recorded three months prior) and the weight at baseline assessment. Weight loss grade (WLG) was also used to account for BMI [23]. Body composition was analyzed using CT scans at the L3 vertebra. When only cervical CT scans were available, an abdominal CT scan at the L3 was concurrently performed. The cross-sectional area of skeletal muscle and adipose tissue were measured and normalized for height (cm^2^/m^2^). Sarcopenia was defined according to the SMI cutoffs proposed by Kubrak et al. [24] for HNSCC (Head and Neck Squamous cell carcinoma) patients (males: ≥45.3 (normal), 45.2–37.5 cm^2^/m^2^ (class I) and <37.5 cm^2^/m^2^ (class II); females: ≥41.0 (normal), 40.9–34.2 (class I) and <34.2 cm^2^/m^2^ (class II)). Myosteatosis was evaluated using mean skeletal muscle attenuation and measured in Hounsfield units, HU. Adipose tissue indices include the intramuscular (IMATI), visceral (VATI), subcutaneous (SATI), and total adipose tissue index (TATI).

Nutritional diagnosis was established using the Patient-Generated Subjective Global Assessment (PG-SGA), validated in Spanish [25]. Nutritional intervention, when indicated, was provided according to ESPEN clinical guidelines.

### 2.5. Muscle Functionality

Muscle functionality was assessed through standardized tests. Hand grip strength, a validated proxy for overall muscular strength, was measured with a Jamar^®^ Plus+ Digital dynamometer (Performance Health Supply, Inc., Cedarburg, WI, USA), and the highest value from three attempts was recorded. Lower-limb strength and endurance were evaluated with the 30 s Sit-to-Stand test, which counted the number of complete chairs rising within the time frame [26]. Functional mobility was measured with the 4 m Gait Speed Test [27]. Global performance status was evaluated using the Eastern Cooperative Oncology Group Performance Status (ECOG PS) [28] and complemented by patient-reported activity level (normal activity, reduced activity and confined to bed/chair > 50% of the day).

### 2.6. Other Biomarkers

HPV status was determined in tumor tissue by double positivity for HPV DNA and p16 immunohistochemistry (p16^INK4a^).

Systemic inflammation was assessed using the modified Glasgow Prognostic Score (mGPS) [29], which integrates serum C-reactive protein (CRP) and albumin concentrations as prognostic indicators in cancer patients [29,30,31,32,33]. The mGPS was defined as follows: CRP > 10 mg/L and albumin < 35 g/L (score = 2); CRP > 10 mg/L with normal albumin (score = 1); and CRP ≤ 10 mg/L (score = 0). The neutrophil-to-lymphocyte ratio (NLR) was also calculated, given its established association with poor survival in HNSCC [34]. CRP, albumin, and complete blood counts were obtained from routine laboratory analyses at diagnosis.

### 2.7. Statistical Analysis

Descriptive statistics summarize baseline characteristics. Group comparisons were performed using χ^2^ or Kruskal–Wallis tests, as appropriate. Bivariate analyses were stratified by HPV status and treatment response. A two-sided *p*-value < 0.05 was considered statistically significant, with multiple comparisons corrected by using the Benjamini–Hochberg method within each HPV-stratified analysis, when applicable. Classification tree analyses were developed for each HPV subgroup using selected pretreatment variables: age, sex, tobacco status, ECOG PS, mGPS, sarcopenia categories (normal, class I and class II), and PG-SGA score as a continuous variable. TNM stage and treatment modality were not included, as the subgroup-specific classification tree analyses were exploratory and focused on a limited set of pretreatment predictors within each HPV stratum. Given the sample size, restricting the number of candidate variables was considered necessary to preserve model stability and interpretability. The minimum terminal node size was set at 10 observations, and a minimum of 20 observations was required to consider node splitting. The complexity parameter was set at 0.001. When appropriate, surrogate splits were used to handle missing values. However, no variable with missing values was retained in the final classification trees; therefore, no surrogate splits were generated. Model performance was optimized through 5-fold cross-validation and pruning, and its performance (area under the curve (AUC), sensitivity, specificity) was estimated within the same resampling framework [35,36]. In the resulting classification trees, the number of patients who achieved CR and those who did not are displayed as absolute counts, together with the percentage of the total cohort represented by each subgroup. All analyses were conducted in R version 4.4.3 (R Foundation for Statistical Computing, Vienna, Austria), and R Studio version 1.4.1106 (Posit Software, PBC, Boston, MA, USA).

## 3. Results

A total of 120 patients were screened, of whom 101 patients were enrolled and completed baseline assessments. During follow-up, four participants were excluded: two withdrew consent, one changed their treatment intent, and one discontinued due to severe treatment toxicity. Thus, 97 patients completed the early post-treatment assessment and were included in the final analysis (Figure 1). The median follow-up time was 5 months [4.6; 6.0].

The study population was balanced by HPV status (51% HPV-positive and 49% HPV-negative) and predominantly male (77%) with a mean age of 59 years (SD 8.2). Baseline characteristics by HPV status are reported in Supplementary Materials Table S1. At diagnosis, HPV-negative patients reported lower energy intake (1325 vs. 1842 kcal; *p* = 0.002), with no significant differences in protein intake between groups according to 24 h food records. Among males, weight loss (%) was higher in HPV-negative patients (6.0% vs. 1.0%; *p* = 0.002), and myosteatosis was more frequent (67% vs. 22%; *p* = 0.001). Nutritional support also differed, with a greater rate of oral/enteral nutrition initiation in HPV-negative patients (60 vs. 20%; *p* < 0.001). PG-SGA scores were higher in HPV-negative patients (9.1 vs. 4.5; *p* < 0.001). Overall, 62% achieved CR while 38% were NCR, with similar proportions across HPV subgroups (Table 1). No significant differences were found in muscle functional test results and inflammatory biomarkers.

### 3.1. Baseline Characteristics According to Treatment Response in HPV-Positive Patients

Among HPV-positive patients, those with NCR had lower baseline weight (69 vs. 88 kg; *p* < 0.001), a higher probability of being classified in WLG 3–4 (47 vs. 13%; *p* = 0.04), lower SMI (49.7 vs. 54.5 cm^2^/m^2^; *p* = 0.013) and reduced SATI (27.0 vs. 52.9 cm^2^/m^2^; *p* = 0.009). They also had elevated PG-SGA scores (6.5 vs. 3.1; *p* = 0.02) and required intensive nutritional support more often (*p* = 0.007). During follow-up, no significant differences in mortality were observed (0 vs. 5%; *p* = 0.38).

### 3.2. Baseline Characteristics According to Treatment Response in HPV-Negative Patients

In HPV-negative patients, NCR was characterized by a higher proportion of ECOG PS 2 (39 vs. 10%; *p* = 0.001), severe WLG (78 vs. 29% in WLG 3–4; *p* = 0.048), lower SMI (45.4 vs. 49.9 cm^2^/m^2^; *p* = 0.013) and higher prevalence of myosteatosis (92 vs. 55%; *p* = 0.050). Follow-up mortality was also higher among NCR (28 vs. 0%; *p* = 0.006).

Across both subtypes, CR was associated with higher VATI (HPV-positive: 63.3 vs. 21.4 cm^2^/m^2^; *p* = 0.048; HPV-negative: 77.8 vs. 16.9 cm^2^/m^2^, *p* = 0.005). Detailed data on body composition, symptom burden, and functional tests are provided in Supplementary Materials Table S2.

### 3.3. Factors Associated with Early Treatment Response

**Figure 2:**
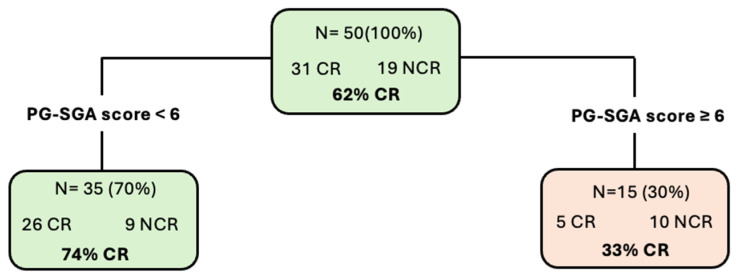
Classification tree of early treatment response from HPV-positive patients.

Classification tree analysis identified PG-SGA score as a relevant factor in the early treatment response in HPV-positive patients (Figure 2). A PG-SGA score < 6 (*n* = 35, 70%) was linked to CR in 74% of cases (26/35), whereas those with PG-SGA scores ≥ 6 (*n* = 15, 30%) had a 33% CR rate (5/15). Model performance showed an AUC of 0.68 (95% CI 0.55–0.82) with 84% sensitivity and 53% specificity. The negative predictive value was 67%.

**Figure 3:**
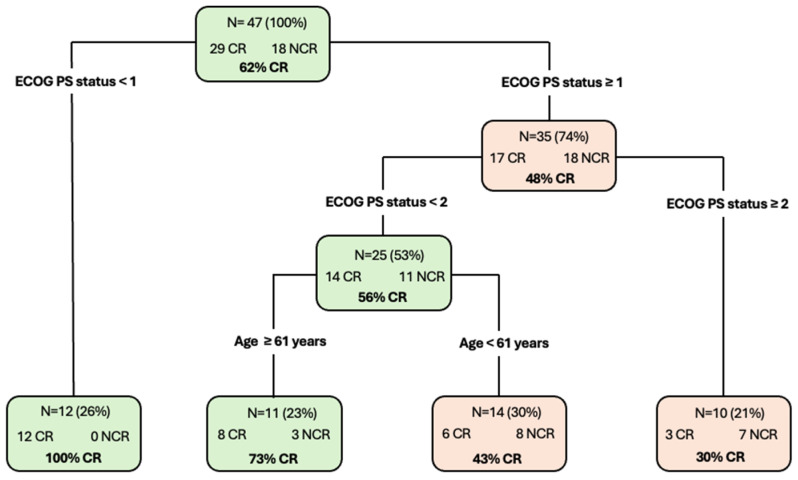
Classification tree of early treatment response from HPV-negative patients.

In HPV-negative patients, ECOG PS and age were the main determinants for early treatment response (Figure 3). All patients with ECOG 0 achieved CR (12/12), while among those with ECOG ≥ 1, CR occurred in 73% of patients aged ≥61 years (8/11) versus 43% of those <61 years (6/14), with ECOG ≥ 2 showing the lowest CR rate (3/10, 30%). The AUC was 0.81 (95% CI 0.70–0.93), with 69% sensitivity and 83% specificity. The negative predictive value was 62%. Complete accuracy metrics are reported in Supplementary Materials Tables S3 and S4. Complementary descriptive analyses indicated that, within the ECOG ≥ 2 subgroup, 9 out of 10 patients presented PG-SGA scores ≥ 9.

Sensitivity analyses using alternative outcome, TNM-stage and treatment-modality weighting strategies yielded the same classification rules as the primary analyses (Supplementary Materials Figures S1 and S2).

## 4. Discussion

Although HPV status is a well-established prognostic factor for long-term outcomes in OPSCC, particularly five-year overall survival [3,37,38], the determinants of early treatment response remain less clearly characterized. In the present study, baseline nutritional status and clinical performance appeared to provide additional information beyond HPV status alone, with distinct response-related profiles emerging according to HPV subgroup. In HPV-positive patients, PG-SGA was the sole variable retained in the classification model, with scores < 6 associated with higher CR rates. These patients also displayed a better baseline nutritional profile, including higher body weight, SMI, and adipose tissue indices. Notably, higher VATI was associated with CR across both HPV subgroups. Although literature specifically addressing early treatment response is limited, prior body-composition studies in HNSCC have linked greater adiposity, including higher visceral adiposity, with more favorable survival outcomes [39]. These results suggest that, even within a biologically favorable HPV-positive population, nutritional status may play a role in early treatment response. In contrast, the early response in HPV-negative patients was primarily related to clinical fitness, as reflected by ECOG performance status and age. In this cohort, all patients with ECOG PS 0 achieved CR, whereas response rates declined markedly with worsening performance status, with only 30% of patients with ECOG PS 2 achieving CR. Complementary analyses further showed that most patients with ECOG PS 2 also had PG-SGA scores ≥ 9, suggesting a substantial overlap between functional impairment and clinically relevant malnutrition. This pattern suggests a broader functional vulnerability profile in HPV-negative disease. Among patients with ECOG PS 1, age further stratified outcomes, with higher CR rates observed in patients aged ≥ 61 years. This finding should be interpreted cautiously, as it may reflect the small size of this subgroup and the exploratory nature of the classification analysis rather than a true independent effect of age. A selection effect also cannot be excluded, whereby older patients with preserved functional status who remained eligible for curative-intent treatment may represent a fitter subgroup. This interpretation is consistent with previous studies suggesting that age alone has limited prognostic value in OPSCC once other host- and tumor-related factors are taken into account [40].

Most previous studies in OPSCC treated with curative CRT have focused on survival, locoregional control, or post-treatment response assessment, and when response has been examined, the emphasis has generally been placed on HPV status, nodal burden, or imaging-based markers rather than on baseline host-related factors [41]. Even when the response has been specifically evaluated, the focus has generally been placed on tumor- and treatment-related variables. In this context, a recent study [42] examined factors associated with other-than-complete response in OPSCC, mainly from the perspective of p16 status, smoking exposure, stage, and tumor subsite. By contrast, our findings suggest that baseline host-related factors provide additional information for early response stratification, and that their relevance differs according to HPV status. Given the reported association between complete response and subsequent outcomes in OPSCC, these findings may contribute to early response stratification.

Malnutrition has consistently been associated with impaired ECOG PS and higher inflammatory burden, both of which contribute to reduced treatment efficacy and poorer survival across cancer types [43,44,45,46]. Few studies have jointly evaluated nutritional diagnosis, CT-based body composition, and functional status as factors associated with early treatment response stratified by HPV status using clinically interpretable classification models.

PG-SGA is a validated tool for nutritional assessment in HNSCC treated with CRT, and has been associated with treatment outcomes [47]. In this cohort, a cutoff of ≥6 points, corresponding to mild to moderate malnutrition, was sufficient to discriminate CR from NCR. This lower threshold compared with those previously reported for toxicity and survival (≥9) [48] suggests that early treatment response may be particularly sensitive to baseline nutritional impairment. From a clinical perspective, PG-SGA is inexpensive, non-invasive and already widely used in nutritional assessment, supporting its incorporation into baseline OPSCC care to identify nutritional risk early, guide closer follow-up and facilitate nutritional support management in line with ESPEN clinical guidelines where appropriate. Beyond global nutritional assessment, micronutrient status may represent another component of nutritional vulnerability not captured in this study. A recent systematic review in HNSCC linked low vitamin D levels to poorer survival and increased treatment-related toxicity, with some evidence suggesting greater prognostic relevance in HPV-negative disease [49]. Although not assessed in our cohort, vitamin D status may therefore be relevant in clinically vulnerable patients, particularly within the HPV-negative subgroup.

Smoking exposure was not retained in either HPV-specific classification model, despite its consistent association with poorer survival outcomes in HPV-positive OPSCC [50]. A similar pattern was observed for sarcopenia and inflammatory biomarkers (mGPS and NLR) after adjustment for PG-SGA or ECOG PS. In this setting, integrative prognostic approaches may be more appropriate. Recent studies [51] demonstrated improved prediction accuracy when clinical, nutritional, immunological, and tumor-related variables—such as TNM stage, p16 status, BMI, the Controlling Nutritional Status (CONUT) score and CD8 expression—are integrated into the same model. Of note, p16 lost significance in this context. Such approaches underscore the complex interplay between nutrition, inflammation, and clinical status in shaping treatment response.

Overall, model discrimination was moderate in HPV-positive patients and higher in HPV-negative patients, despite the lower negative predictive value in the latter, which limits the reliability of NCR prediction in routine clinical practice.

This study has several strengths relevant to clinical translation. Its prospective, multicenter design enabled standardized, real-time data collection across heterogeneous practice settings. Body composition, tobacco exposure and functional status were assessed using validated methodologies, and HPV status was defined by combined p16 immunohistochemistry and HPV DNA testing, maximizing diagnostic specificity. The classification tree approach allowed data-driven identification of clinically interpretable decision rules while accommodating disease heterogeneity. Model performance was evaluated using the AUC, and overfitting was mitigated through cross-validation and pruning. Limiting the analysis to a predefined set of predictors reduced multicollinearity and enhanced interpretability. HPV status and AJCC 8th edition TNM stage were highly collinear within our cohort, and treatment modality showed limited variability across patients, potentially reducing the contribution of these factors to the classification tree analyses. Nevertheless, several limitations warrant consideration. This exploratory analysis was not powered for early response endpoints, as the original cohort was designed to assess survival outcomes. The limited sample size, particularly after HPV stratification, and the underrepresentation of female patients reduced statistical power and increased the risk of chance findings. Moreover, the classification trees were developed within relatively small subgroup samples and should therefore be interpreted as exploratory, cohort-specific classification rules rather than validated prognostic models. The observational design precludes causal inference, and the short follow-up restricts conclusions to early treatment response. Although early treatment response has been associated with subsequent oncologic outcomes, longer follow-up is required to determine whether the factors identified in the present study are also associated with long-term outcomes. Subgroup findings, including age-related effects within ECOG PS 1 patients, should therefore be interpreted cautiously. Despite the use of cross-validation and pruning to reduce overfitting, external validation in independent OPSCC cohorts is required before these findings can be generalized or considered for clinical implementation.

## 5. Conclusions

Baseline nutritional status, as assessed by PG-SGA, emerged as the primary variable associated with early treatment response in HPV-positive patients. In HPV-negative disease, early response was primarily stratified by functional status (ECOG PS) and age; however, the high prevalence of malnutrition in this subgroup suggests a contributory role that may not be fully captured by performance status-based models. Together, these findings support the clinical relevance of integrating nutritional assessment into baseline OPSCC care. External validation in larger cohorts with longer follow-up is warranted to assess implications for survival and long-term disease control.

## Figures and Tables

**Table 1:** Baseline characteristics by treatment response according to HPV status.

Characteristics	HPV-Positive			HPV-Negative		
	CR N = 31	NCR N = 19	Overall *p*-Value	CR N = 29	NCR N = 18	Overall *p*-Value
	N (%)	N (%)		N (%)	N (%)	
Sex			1.00			0.72
Male	22 (71)	14 (74)		24 (83)	14 (78)	
Female	9 (29)	5 (26)		5 (17)	4 (22)	
Age, mean (SD)	57.9 (8.2)	61.0 (8.3)	0.21	61.7 (7.6)	59.9 (8.9)	0.49
Tobacco status			0.06			0.85
Non-regular smoker	6 (19)	1 (5)		1 (3)	1 (6)	
Former smoker (>1 year)	16 (52)	6 (32)		6 (21)	2 (11)	
Smoker	9 (29)	12 (63)		22 (76)	15 (83)	
Alcohol status			0.92			0.28
Non-regular drinker	15 (48)	8 (42)		2 (7)	0 (0)	
Former drinker (>1 year)	4 (13)	2 (11)		8 (28)	9 (50)	
Drinker	12 (39)	9 (47)		19 (66)	9 (50)	
ECOG PS, categories			0.29			**0.001**
0	19 (61)	8 (42)		12 (41)	0 (0)	
1	11 (36)	9 (47)		14 (48)	11 (61)	
2+	1 (3)	2 (11)		3 (10)	7 (39)	
Treatment scheme			**0.03**			**0.10**
CRT	29 (94)	13 (68)		19 (66)	7 (39)	
Induction CT + CRT	1 (3)	4 (21)		9 (31)	8 (44)	
Bio-RT	1 (3)	2 (11)		1 (3)	3 (17)	
Weight (kg), mean (SD)						
Male	88 (14)	69 (13)	**<0.001**	76 (16)	61 (18)	**0.02**
Female	67 (9)	59 (15)	0.30	58 (12)	53 (20)	0.68
WLG categories			**0.04**			**0.048**
0	12 (50)	2 (13)		8 (33)	2 (11)	
1	7 (29)	3 (20)		4 (17)	1 (6)	
2	2 (8)	3 (20)		5 (21)	1 (6)	
3	3 (13)	5 (33)		3 (13)	5 (28)	
4	0 (0)	2 (13)		4 (17)	9 (50)	
Missing	7	4		5	0	
mGPS categories			0.07			0.16
0	23 (92)	13 (72)		18 (72)	8 (47)	
1	1 (4)	5 (28)		5 (20)	8 (47)	
2	1 (4)	0 (0)		2 (8)	1 (6)	
Missing	6	1		4	1	
NLR, median [IQR]	2.0 [1.7; 2.7]	2.6 [1.8; 4.0]	0.28	3.4 [2.6; 5.2]	4.1 [2.4; 4.9]	0.68
SMI grades (Kubrak et al. [24]) in males			1.00			0.29
Normal	19 (95)	10 (91)		15 (75)	7 (54)	
Class I	0 (0)	0 (0)		3 (15)	5 (39)	
Class II	1 (5)	1 (9)		2 (10)	1 (8)	
Missing	11	8		4	1	
SMI grades (Kubrak et al. [24]) in females			0.64			1.00
Normal	4 (44)	0 (0)		1 (25)	0 (0)	
Class I	3 (33)	2 (100)		1 (25)	1 (25)	
Class II	2 (22)	0 (0)		2 (50)	3 (75)	
Missing	0	0		1	0	
PG-SGA score, mean (SD)	3.1 (2.8)	6.5 (5.4)	**0.02**	8.1 (5.4)	10.7 (4.4)	0.08
Nutritional support categories:			**0.007**			1.00
Oral and/or Enteral nutrition	2 (6)	8 (42)		18 (62)	11 (61)	
Dietary counseling + oral supplementation	20 (65)	6 (32)		7 (24)	4 (22)	
Dietary counseling	9 (29)	5 (26)		4 (14)	3 (17)	

Abbreviations: SD: standard deviation; IQR: interquartile range [Q1; Q3]; CR: complete remission; NCR: non-complete remission; ECOG PS: Eastern Cooperative Oncology Group Performance Status; WLG: weight loss grade; NLR: neutrophil-to-lymphocyte ratio; SMI: skeletal muscle index; PG-SGA: Patient-Generated Subjective Global Assessment. mGPS: Modified Glasgow Prognostic Score. Tobacco and alcohol status were categorized as non-regular or former (patients reporting abstinence for >1 year before diagnosis). Bolded *p*-values indicate statistical significance (*p* < 0.05). *p*-values were corrected for multiple comparisons by the Benjamini–Hochberg method.

## Data Availability

The data presented in this study are available on request from the corresponding author due to privacy and ethical restrictions.

## Supplementary Materials

The following supporting information can be downloaded at: https://www.mdpi.com/article/10.3390/nu18132091/s1, Table S1. Clinical, baseline nutritional and body composition characteristics by HPV status. Table S2. Additional clinical, body composition and functional data by treatment response according to HPV status. Table S3. Accuracy measures of the classification tree model in HPV-positive OPSCC patients. Table S4. Accuracy measures of the classification tree model in HPV-negative OPSCC patients. Table S5. Completed STROBE checklist for prospective studies. Figure S1. Sensitivity analysis using equal outcome weighting in HPV-positive and HPV-negative patients. Figure S2. Sensitivity analysis using equal TNM-stage and treatment modality weighting in HPV-positive and HPV-negative patients.

## Abbreviations

The following abbreviations are used in this manuscript:

AUC	Area under the curve
Bio-RT	Radiotherapy with concurrent cetuximab
BMI	Body mass index
C3	Third cervical vertebra
CI	Confidence interval
CO	Carbon monoxide
CONUT	Controlling Nutritional Status
CR	Complete remission
CRP	C-reactive protein
CRT	Concurrent chemoradiotherapy
CT	Computed tomography
ECOG PS	Eastern Cooperative Oncology Group Performance Status
ESPEN	European Society for Clinical Nutrition and Metabolism
FFPE	Formalin-fixed, paraffin-embedded
HNSCC	Head and neck squamous cell carcinoma
HPV	Human papillomavirus
HUB	Hospital Universitari de Bellvitge
HU	Hounsfield units
ICO	Catalan Institute of Oncology
IMATI	Intramuscular adipose tissue index
IQR	Interquartile range
L3	Third lumbar vertebra
mGPS	Modified Glasgow Prognostic Score
NCR	Non-complete remission
NLR	Neutrophil-to-lymphocyte ratio
OPSCC	Oropharyngeal squamous cell carcinoma
PG-SGA	Patient-Generated Subjective Global Assessment
p16^INK4a^	p16 immunohistochemistry
ppm	Parts per million
SATI	Subcutaneous adipose tissue index
SD	Standard deviation
SMI	Skeletal muscle index
STROBE	Strengthening the Reporting of Observational Studies in Epidemiology
TATI	Total adipose tissue index
TNM	Tumor–node–metastasis
VATI	Visceral adipose tissue index
WLG	Weight loss grade
