# Supplementary material for: Baseline Nutritional Status and Early Treatment Response in Oropharyngeal Cancer: A Prospective Cohort Study by HPV Status (FIS 19 Study)
Source: Nutrients. 2026 Jun 26;18(13):2091. doi: 10.3390/nu18132091 (PMC13362776; doi:10.3390/nu18132091)

Supplementary Materials:

Table S1. Clinical, baseline nutritional and body composition characteristics by HPV status.

Table S2. Additional clinical, body composition and functional data by treatment response according to HPV status.

Table S3. Accuracy measures of the classification tree model in HPV-positive OPSCC patients.

Table S4. Accuracy measures of the classification tree model in HPV-negative OPSCC patients.

Table S5. Completed STROBE checklist for prospective studies.

Figure S1. Sensitivity analysis using equal outcome weighting in HPV-positive and HPV-negative patients.

Figure S2. Sensitivity analysis using equal TNM-stage and treatment modality weighting in HPV-positive and HPV-negative patients.

Table S1. Clinical, baseline nutritional and body composition characteristics by HPV status.

Characteristics	HPV-Negative N = 50	HPV-Positive N = 51	Overall *p*-Value
	N (%)	N (%)	
Sex			0.37
Male	41 (82)	37 (73)	
Female	9 (18)	14 (28)	
Age, mean (SD)	61.0 (8.0)	59.2 (8.2)	0.26
CO-oximetry (ppm), mean (SD)	7.3 (8.2)	3.7 (4.7)	**0.009**
Alcohol status			**<0.001**
Non regular drinker	2 (4)	23 (45)	
Former drinker (>1 year)	19 (38)	7 (14)	
Drinker	29 (58)	21 (41)	
TNM			**<0.001**
I/II	0 (0)	22 (43)	
III	5 (10)	29 (57)	
IVa/IVb	45 (90)	0 (0)	
Treatment scheme			**0.006**
CRT	29 (58)	43 (84)	
Induction CT + CRT	17 (34)	5 (10)	
Bio-RT	4 (8)	3 (6)	
Weight (kg), median [IQR]			
Male	70.0 [58.8;82.0]	81.2 [65.3;88.5]	**0.03**
Female	52.0 [43.6;65.7]	63.1 [54.3;73.6]	0.17
Weight loss (%), mean (SD)			
Male	6.0 (8.1)	1.0 (5.2)	**0.002**
Female	11.7 (12.5)	2.7 (9.1)	0.083
WLG categories			**0.047**

0	12 (27)	14 (36)	
1	5 (11)	10 (26)	
2	6 (13)	5 (13)	
3	9 (20)	8 (21)	
4	13 (29)	2 (5)	
Missing	5	12	
mGPS category			**0.03**
0	26 (59)	37 (84)	
1	14 (32)	6 (14)	
2	4 (9)	1 (2)	
Missing	6	7	
NLR, median [IQR]	3.6 [2.5;4.9]	2.1 [1.7;3.2]	**<0.001**
SMI (cm^2/m^2), median [IQR]			
Male	49.1 [42.3;57.0]	53.3 [48.8;57.0]	0.15
Female	32.3 [30.8;36.0]	39.4 [35.4;42.1]	0.083
Missing	6	8	
SMI grades (Kubrak et al.) in male			**0.009**
Normal	25 (69)	29 (91)	
Class I	8 (22)	0 (0)	
Class II	3 (8)	3 (9)	
Missing	5	5	
SMI grades (Kubrak et al.) in female			0.22
Normal	1 (13)	4 (36)	
Class I	2 (25)	5 (46)	
Class II	5 (63)	2 (18)	
Missing	1	3	
Myosteatosis (Martin et al.), yes, in male	24 (67)	7 (22)	**0.001**
Missing	5	5	
Myosteatosis (Martin et al), yes, in female	6 (75)	7 (64)	1.00
Missing	1	3	
IMATI (cm^2/m^2), median [IQR]			
Male	3.5 [2.4;4.5]	2.01 [1.2;3.00]	**0.005**
Female	3.6[1.3;6.2]	3.1 [2.4;3.8]	0.93
Missing	6	8	
VATI (cm^2/m^2), median [IQR]			
Male	66.1 [19.3;92.9]	48.4 [20.9;73.5]	0.40
Female	23.5 [3.0;71.5]	38.3 [18.6;60.3]	0.56
Missing	6	8	
SATI (cm^2/m^2), median [IQR]			
Male	36.6 [25.4;65.3]	44.6 [23.6;64.4]	0.73
Female	35.4 [9.34;74.7]	77.6 [53.6;95.2]	0.10
Missing	6	8	
TATI (cm^2/m^2), median [IQR]			
Male	121 [49.1;155]	105 [46.0;164]	0.63
Female	77.7 [13.7;141]	125 [83.1;143]	0.36
Missing	6	8	
ECOG-PS, categories			**0.02**
0	14 (28)	27 (53)	
1	26 (52)	21 (41)	
2	10 (20)	3 (6)	
Activity categories			**0.004**
Normal activity	18 (36)	30 (59)	
Less than normal	18 (36)	19 (37)	

More than 50% in bed/seated	7 (14)	0 (0)	
Fatigated	7 (14)	2 (4)	
Dynamometer, mean (SD)	31.9 (9.6)	36.4 (10.8)	**0.03**
Sit to stand, median [IQR]	11.0 [9.0;13.2]	12.0 [10.5;14.0]	**0.053**
Missing	2	0	
Gait speed test, median [IQR]	3.7 [3.2;4.8]	3.4 [3.0;3.7]	**0.03**
Missing	2	0	
24-hour Energy intake, median [IQR]	1325 [997;1792]	1841 [1399;2218]	**0.002**
24-hour Protein intake, median [IQR]	60.8 [45.1;81.1]	72.2 [53.5;92.9]	0.10
PG-SGA score, mean (SD)	9.1 (5.1)	4.5 (4.3)	**<0.001**
PG-SGA categories			**<0.001**
A	14 (28)	34 (67)	
B/C	36 (72)	17 (33)	
Nutritional support categories			**<0.001**
Oral and/or Enteral nutrition	30 (60)	10 (20)	
Dietary counselling+ Oral supplementation	12 (24)	27 (53)	
Dietary counselling	8 (16)	14 (28)	

Abbreviations: SD: standard deviation; IQR: interquartile range [Q1; Q3]; CRT: concurrent chemoradiotherapy; Induction CT + CRT: induction chemotherapy followed by chemoradiotherapy; Bio-RT: radiotherapy with concurrent cetuximab; CRP: C-reactive protein; NLR: neutrophil-to-lymphocyte ratio; mGPS: modified Glasgow Prognostic Score; SMI: skeletal muscle index; IMATI: intramuscular adipose tissue index; VATI: visceral adipose tissue index; SATI: subcutaneous adipose tissue index; TATI: total adipose tissue index; WLG: weight loss grade; PG-SGA: Patient-Generated Subjective Global Assessment. Alcohol status was categorized as current, non-regular, or former (patients reporting abstinence for >1 year before diagnosis). Bolded p-values indicate statistical significance (p < 0.05).

Table S2. Additional clinical, body composition and functional data by treatment response according to HPV status.

Characteristics	HPV-Positive			HPV-Negative		
	CR N = 31		Overall *p*-Value	CR N = 29	NCR N = 18	Overall *p*-Value
	N (%)	N (%)		N (%)	N (%)	
CO-oximetry (ppm), median [IQR]	2.0 [1.0;3.0]	2.0 [1.0;8.0]	0.33	5.5 [2.0;10.0]	4.0 [2.0;7.5]	0.72
Myosteatosis (Martin et al), yes, in male	2 (10)	4 (36)	0.15	11 (55)	12 (92)	**0.050**
Missing	2	3		4	1	
Myosteatosis (Martin et al), yes, in female	5 (56)	2 (100)	0.49	4 (100)	2 (50)	0.43
Missing	0	3		1	0	
IMATI (cm²/m²), median [IQR]						
Male	2.0 [1.3;2.6]	1.5 [1.0;3.1]	0.68	3.3 [2.2;4.2]	3.7 [2.8;4.3]	0.85
Female	3.1 [2.3; 4.2]	2.9 [2.7; 3.2]	1.00	3.6 [2.4;4.8]	4.3 [0.6;11.7]	1.00
Missing	2	6		5	1	
VATI (cm²/m²), median [IQR]						
Male	63.3 [41.5;83.5]	21.4 [11.4;57.6]	**0.048**	77.8 [47.2;100]	16.9 [13.6;47.1]	**0.005**
Female	38.3 [27.5;63.4]	28.9 [19.3;38.5]	0.64	23.2 [3.04;49.5]	41.5 [3.3;80.4]	0.56
Missing	2	6		5	1	
SATI (cm²/m²), median [IQR]						
Male	52.9 [37.3;88.6]	27.0 [12.1;45.6]	**0.009**	38.9 [27.9;67.1]	24.6 [12.2;41.2]	0.08
Female	79.0 [66.2;99.3]	43.7 [36.5;50.9]	0.16	42.4 [8.88;74.7]	35.4 [18.9;73.0]	0.77
Missing	2	6		5	1	
TATI (cm²/m²), median [IQR]						
Male	117 [90.3;173]	49.0 [24.3;105]	**0.02**	127 [94.0;181]	47.4 [28.0;92.0]	**0.03**
Female	129 [108;146]	75.5 [58.9;92.1]	0.24	71.5 [13.7;132]	84.1 [22.8;168]	0.77

Missing	2	6		5	1	
Activity categories			1.00			0.11
Normal activity	18 (58)	11 (58)		11 (38)	5 (28)	
Less than normal	12 (39)	7 (37)		13 (45)	5 (28)	
>50% in bed/seated	1 (3)	1 (5)		4 (13.8)	3 (17)	
Fatigated	0 (0)	0 (0)		1 (3)	5 (28)	
Dynamometer, mean (SD)	37.0 (10.9)	35.2 (11.2)	0.58	33.8 (9.5)	28.5 (9.1)	0.06
Sit to stand, mean (SD)	12.0 (3.6)	13.3 (5.4)	0.39	11.1 (3.5)	11.4 (2.8)	0.78
Gait speed test, mean (SD)	3.6 (1.8)	3.9 (1.5)	0.54	4.30 (1.9)	4.21(1.9)	0.88
Anorexia, yes	6 (19)	5 (26)	0.73	11 (38)	7 (39)	1.00
Dysphagia, yes	7 (23)	9 (47)	0.13	12 (41)	12 (67)	0.17
Odynophagia, yes	11 (36)	7 (37)	1.00	18 (62)	13 (72)	0.69
Dental problems, yes	1 (3)	4 (21)	0.06	2 (7)	7 (39)	**0.02**

Abbreviations: CR: Complete remission; NCR: Non-complete remission; IMATI: Intramuscular Adipose Tissue Index; VATI: Visceral Adipose Tissue Index; SATI: Subcutaneous Adipose Tissue Index; TATI: Total Adipose Tissue Index. *Bolded p-values indicate statistical significance ($p < 0.05$). IQR: Q1; Q3. P-values are corrected for multiple comparisons by Benjamini–Hochberg method.

Table S3. Accuracy measures of the classification tree model in HPV-positive OPSCC patients.

Parameter	Estimate (95% CI)
Apparent prevalence	0.70 (0.55, 0.82)
True prevalence	0.62 (0.47, 0.75)
Sensitivity	0.84 (0.66, 0.95)
Specificity	0.53 (0.29, 0.76)
Positive predictive value	0.74 (0.57, 0.88)
Negative predictive value	0.67 (0.38, 0.88)
Positive likelihood ratio	1.77 (1.08, 2.91)
Negative likelihood ratio	0.31 (0.12, 0.76)
Correctly classified proportion	0.72 (0.58, 0.84)

Apparent prevalence, patients classified as CR; True prevalence, patients with actual CR; Sensitivity, proportion of true CR correctly identified; Specificity, proportion of non-CR correctly identified; Positive predictive value, probability that predicted CR is true CR; Negative predictive value, probability that predicted NCR is true NCR; Positive likelihood ratio, odds of CR if predicted CR; Negative likelihood ratio, odds of CR if predicted NCR; Correctly classified proportion, overall accuracy of the model.

Table S4. Accuracy measures of the classification tree model in HPV-negative OPSCC patients.

Parameter	Estimate (95% CI)
Apparent prevalence	0.49 (0.34, 0.64)
True prevalence	0.62 (0.46, 0.75)
Sensitivity	0.69 (0.49, 0.85)
Specificity	0.83 (0.59, 0.96)
Positive predictive value	0.87 (0.66, 0.97)
Negative predictive value	0.62 (0.41, 0.81)
Positive likelihood ratio	4.14 (1.43, 11.96)
Negative likelihood ratio	0.37 (0.21, 0.67)
Correctly classified proportion	0.74 (0.60, 0.86)

Apparent prevalence, patients classified as CR; True prevalence, patients with actual CR; Sensitivity, proportion of true CR correctly identified; Specificity, proportion of non-CR correctly identified; Positive predictive value, probability that predicted CR is true CR; Negative predictive value, prob-

ability that predicted NCR is true NCR; Positive likelihood ratio, odds of CR if predicted CR; Negative likelihood ratio, odds of CR if predicted NCR; Correctly classified proportion, overall accuracy of the model.

Table S5. Completed STROBE checklist for prospective studies.

	Item No.	Recommendation	Page No.	Relevant text from manuscript
**Title and abstract**	1	(*a*) Indicate the study's design with a commonly used term in the title or the abstract	1-2	This study is based on a prospective recruitment of a multicenter observational cohort, with retrospective analysis of the collected data.
		(*b*) Provide in the abstract an informative and balanced summary of what was done and what was found	1-2	The abstract includes all this information.
**Introduction**				
Background/rationale	2	Explain the scientific background and rationale for the investigation being reported	2-3	The introduction describes how HPV status is traditionally considered a major prognostic factor in OPSCC, but recent evidence suggests that nutritional, body composition, inflammatory and toxic habits may also significantly influence treatment outcomes. The study aims to clarify the role of these factors in early treatment response.
Objectives	3	State specific objectives, including any prespecified hypotheses	3	Aim: Assess the predictive and prognostic value of smoking status, body composition, nutritional status and inflammatory biomarkers in patients with OPSCC according to its HPV status.<br>Hypothesis: Body composition, nutritional status and inflammatory markers predict better the early treatment response of OPSCC patients
**Methods**				
Study design	4	Present key elements of study design early in the paper	3-4	Prospective, multicenter observational cohort study, detailed in Section 2.1.
Setting	5	Describe the setting, locations, and relevant dates, including periods of recruitment, exposure, follow-up, and data collection	3-4	Conducted at Catalan Institute of Oncology (ICO)-Hospitalet and Bellvitge Hospital (HUB), ICO-Badalona and Hospital del Mar. Patients were recruited between 12/21 and 06/24. Data were collected at diagnosis.
Participants	6	(*a*) *Cohort study*—Give the eligibility criteria, and the sources and methods of selection of participants. Describe methods of follow-up	3-4	Inclusion criteria: adults with histologically confirmed OPSCC, eligible for curative-intent treatment, and available baseline data. Exclusion: metastatic disease, prior head and neck cancer treatment. Follow-up involved scheduled clinical assessments and imaging to evaluate early treatment response.
		(*b*) *Cohort study*—For matched studies, give matching criteria and number of exposed and unexposed		Not applicable
Variables	7	Clearly define all outcomes, exposures, predictors, potential confounders, and effect modifiers. Give diagnostic criteria, if applicable	4-5	Outcome: Early treatment response, classified as complete remission (CR) or non-complete remission (NCR) assessed by clinical and imaging criteria.<br>Main predictors: Nutritional, body composition, functional and clinical variables described in methodology Section 2.<br>Potential confounders: Given the exploratory nature of the study and the limited sample size within HPV-

				stratified analyses, classification tree models were restricted to a predefined set of pretreatment candidate predictors to preserve model stability and interpretability.<br>Effect modifier: HPV status, determined by double positivity for HPV DNA and p16^{INK4a}
Data sources/ measurement	8	For each variable of interest, give sources of data and details of methods of assessment (measurement). Describe comparability of assessment methods if there is more than one group	4-5	Nutritional status assessed using validated clinical tools. Body composition measured by CT scan at L3 level. Functional performance assessed using 30-second sit-to-stand test. Smoking/alcohol history by structured patient interviews and objectively measured using Co-oximetry. HPV status via p16^{INK4a} and HPV DNA PCR. All assessments were standardized across centers.
Bias	9	Describe any efforts to address potential sources of bias	5-6	Standardized protocols for data collection across sites reduced measurement bias. Exclusion of metastatic cases ensured homogeneity of disease stage. Use of validated instruments for data collection. Validated instruments were used for nutritional, functional and body composition assessments. Classification tree models were stratified by HPV status and optimized using repeated cross-validation and pruning to reduce overfitting.
Study size	10	Explain how the study size was arrived at	4	No priori sample size calculation was performed for this exploratory endpoint.
Quantitative variables	11	Explain how quantitative variables were handled in the analyses. If applicable, describe which groupings were chosen and why	5-6	Continuous variables including weight loss (%), 24-hour energy intake (kcal), serum albumin (g/L), and VATI were analyzed both as continuous measures and as categorical variables. The categorization thresholds were derived from decision tree analyses, which allowed for data-driven cut-off selection based on optimal discrimination of treatment response outcomes.
Statistical methods	12	(*a*) Describe all statistical methods, including those used to control for confounding	5-6	Baseline characteristics were compared with χ^2 or Kruskal–Wallis tests, with Benjamini–Hochberg adjustment for multiple testing. Early treatment response predictors were evaluated using classification trees fitted separately by HPV status with seven pre-treatment variables (age, sex, tobacco status, ECOG PS, PG-SGA score, SMI by Kubrak categories, mGPS). Model performance was optimized using repeated cross-validation and pruning, and performance measures (AUC, sensitivity and specificity) were estimated within the same resampling framework.
		(*b*) Describe any methods used to examine subgroups and interactions	5-6	Exploratory subgroup analyses were performed according to HPV status using separate classification tree analyses in HPV-positive and HPV-negative patients.
		(*c*) Explain how missing data were addressed	5-6	Cases with missing data on the primary outcome or on key predictors variables were excluded from the analyses. For categorical variables, a separate "missing" category was created to retain these cases in

				the models. No imputation procedures were performed. Surrogate splits were available to accommodate missing observations in the classification tree analyses; however, no variable with missing values was retained in the final classification trees, and therefore surrogate splits were not required.
		(*d*) *Cohort study*—If applicable, explain how loss to follow-up was addressed	5-6	Minimal loss to follow-up occurred given the short observation window for early response. All included patients completed baseline and post-treatment assessments.
		(*e*) Describe any sensitivity analyses	5-6	Sensitivity analyses using alternative outcome and TNM-stage weighting strategies were conducted and yielded the same classification rules as the primary analyses (Supplementary Figures S1 and S2).
**Results**				
Participants	13	(a) Report numbers of individuals at each stage of study—eg numbers potentially eligible, examined for eligibility, confirmed eligible, included in the study, completing follow-up, and analysed	6	A total of 120 patients were assessed; 104 met inclusion criteria and were enrolled. After exclusions for missing data, 101 were included in the final analysis. From the included participants, 97 completed the follow-up for early treatment response.
		(b) Give reasons for non-participation at each stage	6	Reasons for exclusion included metastatic disease, prior cancer treatment, withdrawal of consent, treatment intent modification, and severe treatment toxicity. Participant flow is summarized in Figure 1.
		(c) Consider use of a flow diagram	6	A flowchart was included in the manuscript (Figure 1) detailing the enrollment and follow-up process.
Descriptive data	14	(a) Give characteristics of study participants (eg demographic, clinical, social) and information on exposures and potential confounders	6-8	Patient descriptives are given by HPV status (Tables S1 and S2) and stratified by both HPV status and treatment response that are shown in Table 1 a/b and table 2 a/b. Everything is described in the results (Section 3).
		(b) Indicate number of participants with missing data for each variable of interest	Tables	No missing data in clinical and nutritional data at baseline. Post-treatments were N =10 missing's. Body composition: N = 14 patients without available or good quality PET/TC scan in baseline.
		(c) *Cohort study*—Summarise follow-up time (eg, average and total amount)		Median of 5 months from the diagnosis date to the treatment response evaluation (two months after treatment).
Outcome data	15	*Cohort study*—Report numbers of outcome events or summary measures over time	6-8	Of the 97 patients included in the final analysis (median follow-up 5 months [4.6–6.0]), 61.8% achieved complete remission (CR) and 38.2% had non-complete remission (NCR), with similar proportions across HPV subgroups. Among HPV-positive patients, NCR was associated with lower baseline weight, higher WLG, lower SMI, reduced SATI and higher PG-SGA scores. In HPV-negative patients, NCR was associated with ECOG PS 2, severe WLG, lower SMI and higher prevalence of myosteatosis. Detailed outcome measures according to HPV status are reported in Table 1 and Supplementary Table S2.

Main results	16	(*a*) Give unadjusted estimates and, if applicable, confounder-adjusted estimates and their precision (eg, 95% confidence interval). Make clear which confounders were adjusted for and why they were included	7-9	Classification tree analyses identified distinct response-associated profiles according to HPV status. In HPV-positive patients, PG-SGA was the sole variable retained in the final model, with patients scoring <6 achieving CR more frequently than those scoring ≥6. Model performance showed an AUC of 0.68 (95% CI 0.55–0.82), with 84% sensitivity and 53% specificity. In HPV-negative patients, ECOG-PS and age were the variables retained in the final model. Model performance showed an AUC of 0.81 (95% CI 0.70–0.93), with 69% sensitivity and 83% specificity.
		(*b*) Report category boundaries when continuous variables were categorized	7-9	Continuous variables were categorized using cut-offs identified via decision tree modeling. Data-driven category boundaries identified by the models included PG-SGA score (<6 vs ≥6) in HPV-positive patients and age (<61 vs ≥61 years) among HPV-negative patients with ECOG ≥1.
		(*c*) If relevant, consider translating estimates of relative risk into absolute risk for a meaningful time period		Not applicable
Other analyses	17	Report other analyses done—e.g., analyses of subgroups and interactions, and sensitivity analyses	7-9	Exploratory subgroup analyses stratified by HPV status were performed. Sensitivity analyses were performed by (i) assigning equal weight to both outcome categories and (ii) assigning equal weight across TNM stage and treatment categories to assess the robustness of the classification models.
**Discussion**				
Key results	18	Summarise key results with reference to study objectives	9-10	Early treatment response was mainly predicted by nutritional status in HPV-positive patients and by functional status and age in HPV-negative patients. Model performance was moderate in HPV-positive and higher in HPV-negative patients.
Limitations	19	Discuss limitations of the study, taking into account sources of potential bias or imprecision. Discuss both direction and magnitude of any potential bias	9-10	Limitations include modest sample size, observational design (no causality), potential for residual confounding, use of categorical cut-offs, and restricted follow-up to early response.
Interpretation	20	Give a cautious overall interpretation of results considering objectives, limitations, multiplicity of analyses, results from similar studies, and other relevant evidence	9-10	These findings suggest that nutritional and functional factors may provide additional information for early response stratification in OPSCC. However, the exploratory nature of the analysis and the limited sample size warrant cautious interpretation and external validation.
Generalisability	21	Discuss the generalisability (external validity) of the study results		The multicenter design enhances generalisability to real-world clinical care. However, external validation is needed with a larger cohort of patients.
**Other information**				
Funding	22	Give the source of funding and the role of the funders for the present study and, if applicable, for the original study on which the present article is based	9	The study was supported by Instituto de Salud Carlos III through the grant FI20/00103, which is co-founded by the European Social Foundation (ESF). The funders had no role in study design, data collection, analysis, interpretation, or manuscript preparation.

This checklist was elaborated using formal items recommended for prospective studies from STROBE statement (https://www.strobe-statement.org).

Figure S1. Sensitivity analysis using equal outcome weighting in HPV-positive and HPV-negative patients.

HPV-Positive

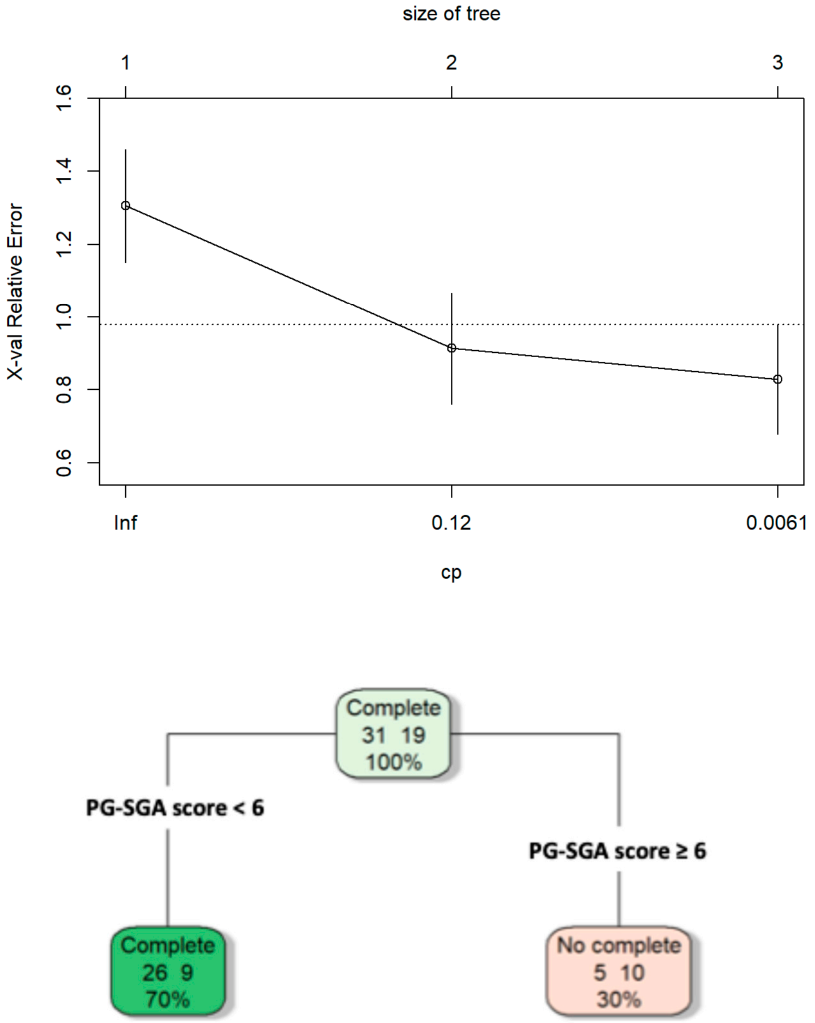

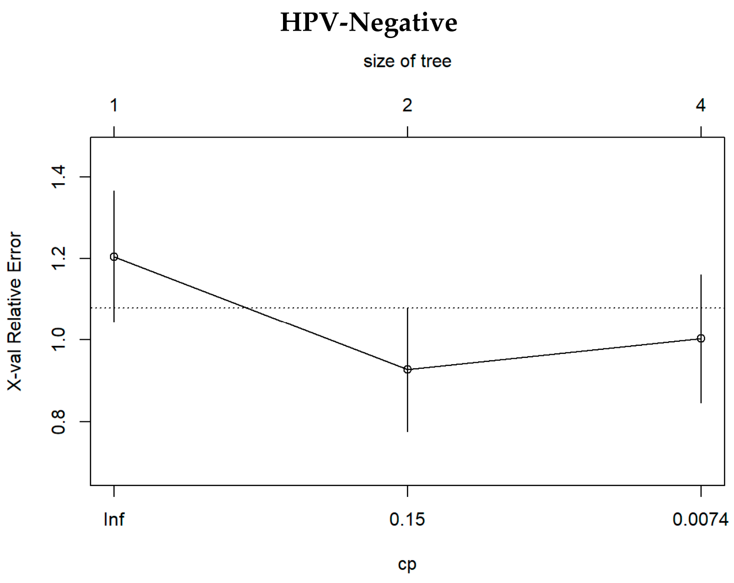
HPV-Negative
size of tree
1
2
4
X-val Relative Error
0.8
1.0
1.2
1.4
Inf
0.15
0.0074
cp

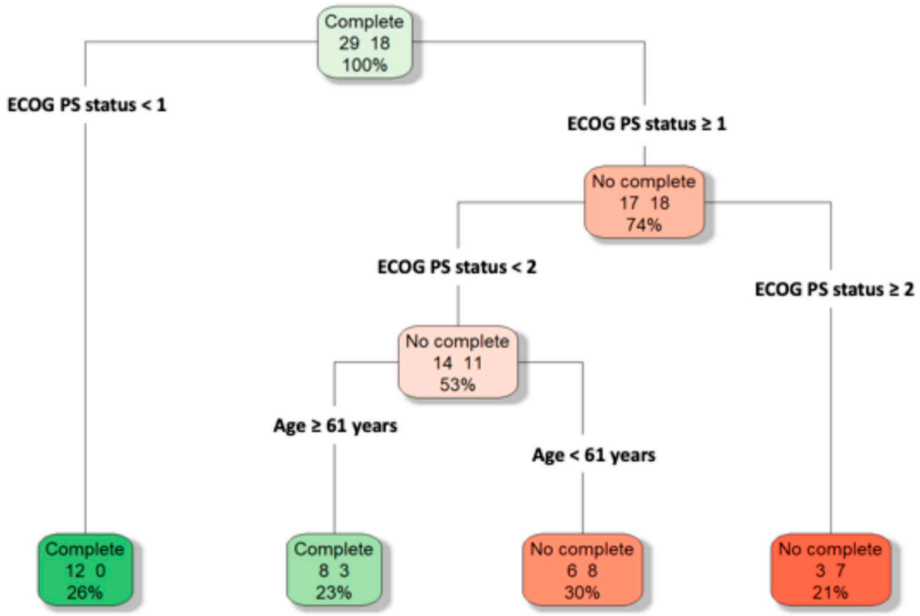
Complete
29 18
100%
ECOG PS status < 1
ECOG PS status ≥ 1
No complete
17 18
74%
ECOG PS status < 2
ECOG PS status ≥ 2
No complete
14 11
53%
Age ≥ 61 years
Age < 61 years
Complete
12 0
26%
Complete
8 3
23%
No complete
6 8
30%
No complete
3 7
21%

Figure S2. Sensitivity analysis using equal TNM-stage and treatment modality weighting in HPV-positive and HPV-negative patients.

HPV-Positive

With CRT/CT induction equal weights

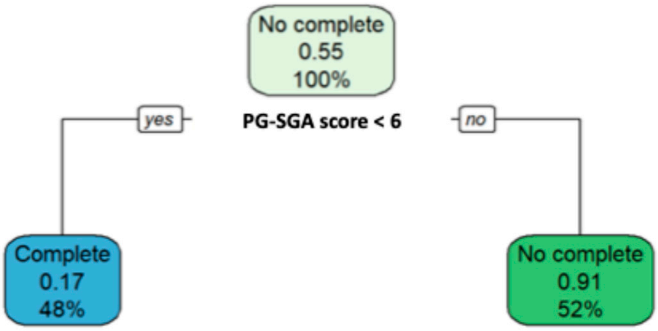

TNM 8th ed equal weights

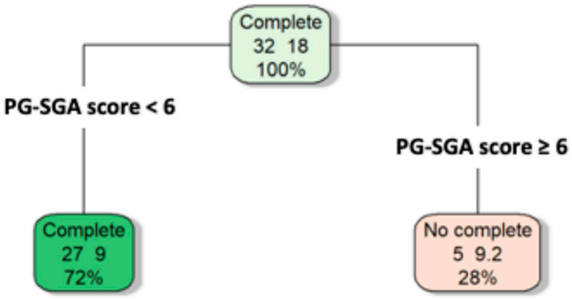

HPV-Negative

With CRT/CT induction equal weights

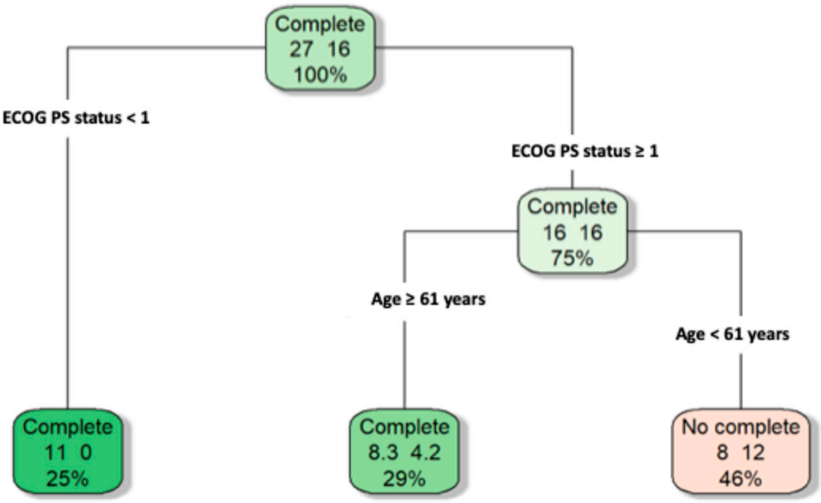

TNM 8th ed equal weights (IVa vs IVb)

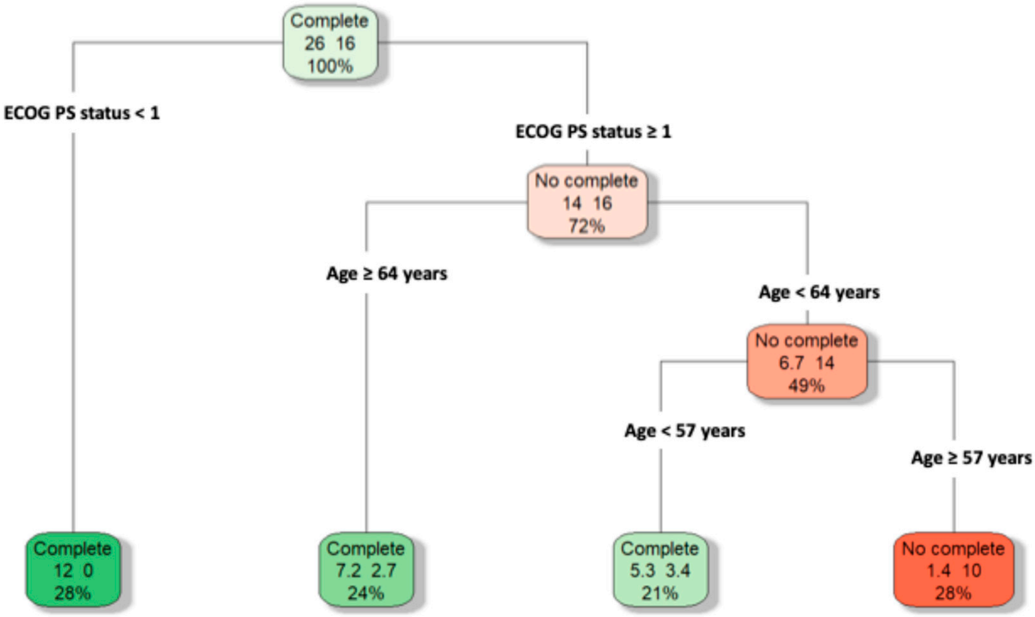

Supplement: Supplementary file 1 [file nutrients-18-02091-s001.zip › nutrients-4364512-supplementary.pdf]
